# Conceptual Examination of Pt Atom-Adorned WTe_2_ for Improved Adsorption and Identification of CO and C_2_H_4_ in Dissolved Gas Analysis

**DOI:** 10.3390/ma17225487

**Published:** 2024-11-10

**Authors:** Qi Zhao, Suya Li, Jin He, Yuyan Man, Songyuan Li

**Affiliations:** 1 State Grid Tianjin Electric Power Research Institute, Tianjin 300384, China; 2 Tianjin Key Laboratory of Things in Electricity, Tianjin 300384, China; 3 State Grid Tianjin Electric Power Company, Tianjin 300232, China

**Keywords:** density functional theory, WTe_2_ monolayer, TM atom, dissolved gas, adsorption, sensor

## Abstract

The online monitoring of transformer insulation is crucial for ensuring power system stability and safety. Dissolved gas analysis (DGA), employing highly sensitive gas sensors to detect dissolved gas in transformer oil, offers a promising means to assess equipment insulation performance. Based on density functional theory (DFT), platinum modification of a WTe_2_ monolayer was studied and the adsorption behavior of CO and C_2_H_4_ on the Pt-WTe_2_ monolayer was simulated. The results showed that the Pt atom could be firmly anchored to the W atoms in the WTe_2_ monolayer, with a binding energy of −3.12 eV. The Pt-WTe_2_ monolayer showed a trend toward chemical adsorption to CO and C_2_H_4_ with adsorption energies of −2.46 and −1.88 eV, respectively, highlighting a stronger ability of Pt-WTe_2_ to adsorb CO compared with C_2_H_4_. Analyses of the band structure (BS) and density of states (DOS) revealed altered electronic properties in the Pt-WTe_2_ monolayer after gas adsorption. The bandgap decreased to 1.082 eV in the CO system and 1.084 eV in the C_2_H_4_ system, indicating a stronger interaction of Pt-WTe_2_ with CO, corroborated by the analysis of DOS. Moreover, the observed change in work function (WF) was more significant in CO systems, suggesting the potential of Pt-WTe_2_ as a WF-based gas sensor for CO detection. This study unveils the gas-sensing potential of the Pt-WTe_2_ monolayer for transformer status evaluation, paving the way for the development of gas sensor preparation for DGA.

## 1. Introduction

In the realm of power supply systems, oil-soaked power transformers comprise around 90%, holding paramount importance in ensuring efficient power distribution. However, these transformers are prone to internal faults, such as overheating or partial discharge during prolonged operation. These faults generate gas molecules (H_2_, CH_4_, C_2_H_2_, etc.) [1,2,3], leading to the dissolution of these oil decomposition products into the transformer oil. The process in question has the potential to compromise the insulation performance of the oil, which could have significant implications for the safe operation of oil-soaked transformers [4,5]. Detecting dissolved gases in transformer oil holds immense significance in averting deterioration accidents and conserving maintenance investment for equipment. Dissolved gas analysis (DGA) serves as a highly effective method for assessing transformer operational conditions by leveraging gas generation phenomena during internal faults [6,7]. The technique entails the extraction of dissolved gases from the transformer oil, followed by the utilization of conventional gas sensors for the identification of the extracted gases. Among diverse detection methods, resistive sensors have attracted substantial interest because of their high sensitivity, rapid response, and recovery times in gas detection [8,9]. Following decades of development, this approach has evolved into a viable and effective method for evaluating the operational status of oil-immersed transformers [10].

Two-dimensional (2D) materials are widely considered in gas sensing applications owing to their expansive larger surface area, higher carrier mobility, distinctive electronic properties, and low energy consumption. Since graphene’s discovery in 2004 [11], researchers have extensively explored the gas-sensing capabilities of 2D materials. More recently, layered transition metal dichalcogenides (TMDs) represented by the molecular formula MX_2_ (M = Zr, W, etc., X = Te, Se, etc.) have emerged as focal points in the field of 2D materials, showcasing noticeable characteristics [12,13,14,15,16]. Among these materials, ZrTe_2_, WTe_2_, WSe_2_, and NiTe_2_ are particularly intriguing members within the TMD family. The individual MX_2_ layers are held together by robust covalent bonds, while weak van der Waals forces maintain interlayer bonds [17,18]. This distinctive bonding structure gives rise to various captivating physical properties, rich intercalation chemistry, and numerous potential applications [19,20]. TMDs commonly exhibit hexagonal symmetry and space groups (SG) P-3m1 and P63/mmc, which permit the intercalation of other atomic species and complexes within the region between adjacent chalcogen planes (van der Waals gap) [21,22,23,24,25,26]. Furthermore, researchers have investigated the potential of TMDs in other domains, including photocatalysis, optoelectronics, and photovoltaics [27,28,29,30,31].

Nowadays, TMDs, a category of innovative 2D nanomaterials, have been subjected to extensive study with a view to their potential use in gas-sensing applications, both experimentally and theoretically [32,33,34,35,36]. Specifically, they have been proposed for detecting typical gases (C_2_H_2_ and C_2_H_4_) present in transformer oil, including MoS_2_ [37], SnS_2_ [38] and MoTe_2_ [39]. In addition to pioneering TMDs, researchers in the field of gas sensing are engaged in the ongoing exploration of novel TMDs based on different transition metals (TMs); in particular, WX_2_ (X = S, Se, Te) has exhibited gas-sensing performances comparable to MoX_2_ [40,41]. For example, WS_2_ and WSe_2_ have exhibited excellent adsorption and sensing properties of SF_6_-decomposed compounds [42,43,44]. Similar to other TMDs, WTe_2_ possesses a substantial surface area, high carrier mobility, and satisfactory dynamic stability [45], laying crucial groundwork for its utilization as a gas sensor. Sarvazaed et al. [46] conducted a DFT analysis on the dynamics between hydrogen and Na/Li-doped WTe_2_, revealing that Li-doped WTe_2_ has higher adsorption energy compared with Na-doped WTe_2_, suggesting its suitability for hydrogen sensing. Scholars tend to modify the physicochemical properties of sensors to enhance their adsorption stability. Significantly, Pt, identified as a TM element, is frequently employed as a dopant to markedly enhance WX_2_‘s adsorption efficiency owing to its robust catalytic properties and electron movement. Nonetheless, research focusing on the adsorption process of Pt-WTe_2_ in detecting gases has been scarce. Consequently, exploring improved detection methods for doping TM atoms in the WTe_2_ monolayer presents significant opportunities for future studies.

This research explores theoretically the use of Pt-adorned WTe_2_ in sensing dissolved gases, and CO and C_2_H_4_ are selected as the research objects to facilitate comparison with the experimental results of other materials to verify the performance of WTe_2_ materials as the physical and chemical properties of CO and C_2_H_4_ are relatively stable, and the adsorption behavior of CO and C_2_H_4_ has been studied on a variety of two-dimensional materials. Utilizing DFT calculations, we pinpointed the best adsorption configurations for unaltered WTe_2_ and Pt-WTe_2_. We concentrated our investigation on their absorption patterns, covering aspects like adsorption energies, electron movement, distance of adsorption, and magnetic attributes. Furthermore, our analysis encompassed electron distribution, band structure (BS), and density of states (DOS) for more profound understanding. For assessing sensing characteristics, the work function (WF), desorption tendencies, and electrical conductivity of Pt-WTe_2_ were computed. This study provides extensive insight into the improved adsorption and detection abilities of WTe_2_ monolayers through the doping of single Pt atoms. Additionally, our findings highlight the considerable capabilities of WTe_2_-based gas sensors in identifying CO and C_2_H_4_. The results underscore the efficacy of altering a single TM atom in surface engineering to enhance WTe_2_’s gas detection abilities.

## 2. Methods

Every spin-polarized DFT computation was performed utilizing the DMol^3^ software [47,48]. To characterize the exchange-correlation function, the Perdew–Burke–Ernzerhof (PBE) functional’s generalized gradient approximation (GGA) technique was selected [49,50,51]. The Tkatchenko–Scheffler technique was employed [52] to consider the van der Waals forces between the gas and the detection material. Utilizing a double numerical polarization (DNP) basis set, the cutoff radii were established at 5.0 Å, achieved through DFT semi-core pseudopots. For the purpose of geometric optimization, the Monkhorst-Pack k value was established at 10 × 10 × 1. Convergence standards for successive phases of geometric optimization were established at 10^−5^ Ha (1 Ha = 27.21 eV) for energy variance, 0.002 Ha/Å for peak force, and 0.005 Å for utmost displacement.

**Figure 1:**
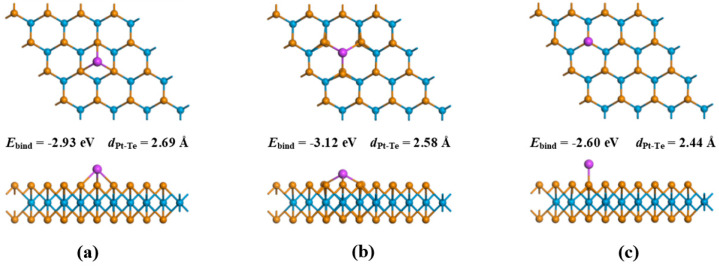
(**a**) Pt-WTe_2_ (T_H_); (**b**) Pt-WTe_2_ (T_W_); (**c**) Pt-WTe_2_ (T_Te_).

Three modification sites for the Pt atom onto pristine WTe_2_ were considered, identified as SH (above the center of the hexagonal ring of WTe_2_), SW (right above the W atom), and STe (right above the Te atom), as illustrated in Figure 1. To assess the strength of the bond between a single Pt atom and the WTe_2_ monolayer, we defined the binding energy (*E_B_*) as follows:

(1) $$E_{B}=E_{{Pt\text{-}WTe}_{2}}-E_{{WTe}_{2}}-E_{Pt\;atom}$$

where $E_{{Pt\text{-}WTe}_{2}}$, $E_{{WTe}_{2}}$, and $E_{Pt\;atom}$ are the calculated total energies of single Pt atom-doped WTe_2_, pristine WTe_2_, and single Pt atom, respectively. The structure with the largest *E_B_* was the most favorable doping site, and gas adsorption was carried out on its surface.

To calculate the adsorption of the gas molecule on Pt-WTe_2_, the adsorption energy was defined as follows:

(2) $${\;E}_{ad}=E_{{gas\;on\;Pt\text{-}WTe}_{2}}-E_{{Pt\text{-}WTe}_{2}}-E_{gas}\;$$

where $E_{{gas\;on\;Pt\text{-}WTe}_{2}}$ indicates the energy of the system after adsorption, $E_{gas}$ and $E_{{Pt\text{-}WTe}_{2}}$ are the total energies of the gas molecule and the Pt-WTe_2_ monolayer before adsorption, and Hirshfeld analysis [53] was employed to determine the electrical charge of each atom across all systems. The charge transfer between gas molecules and the sensing material can be determined by the Hirshfeld (HI) method. The calculation formula is as follows:

(3) $$Q=\int \left(\frac{\rho_{o}\left(\tau \right)}{\sum \rho_{o}^{\prime }\left(\tau \right)}(\rho \left(\tau \right)-\sum \rho_{o}^{\prime }\left(\tau \right))\right)d\tau \;$$

where $\rho \left(\tau \right)$ is the electron density of isolated atom, $\sum \rho_{o}^{'}\left(\tau \right)$ is the sum of the electron density of every atom, and *ρ*(*τ*) is the electron density of the overall structure. This method can accurately evaluate electron transfer. When the value of electron transfer is negative, it indicates that the electrons tend to gather near the gas molecule, and the gas molecule shows negative charge. Furthermore, for an in-depth analysis of electron movement between the gas molecule and Pt-WTe_2_ monolayer, the charge difference density (CDD) was determined in the following manner:

(4) $$∆\rho =\rho_{{gas\;on\;Pt\text{-}WTe}_{2}}-\rho_{gas}-\rho_{{Pt\text{-}WTe}_{2}}\;$$

where $\rho_{{gas\;on\;Pt\text{-}WTe}_{2}}$, $\rho_{{Pt\text{-}WTe}_{2}}$, and $\rho_{gas}$ are the electron densities of the system after adsorption, the Pt-WTe_2_ before adsorption, and the gas molecule.

## 3. Results

### 3.1. Structure of Pristine WTe_2_ and Pt-WTe_2_

**Figure 2:**
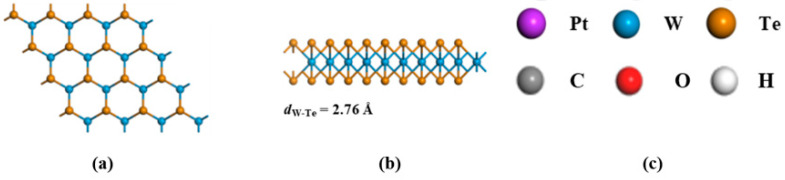
(**a**) Top view of the WTe_2_ monolayer; (**b**) side view of the WTe_2_ monolayer; (**c**) atom specification.

Our initial step involved creating and refining the geometric configuration of the WTe_2_ monolayer, as depicted in Figure 2, leading to a W-Te bond length of 2.76 Å. Following this, we altered the WTe_2_ monolayer’s surface by incorporating a single Pt atom. Taking into account the three alteration points shown in Figure 1, the determined binding energies for the Pt-WTe_2_ formations were −2.93 eV, −3.12 eV, and −2.60 eV, aligning with Figure 1a, Figure 1b, and Figure 1c respectively. Remarkably, the Pt-WTe_2_ configuration shown in Figure 1b demonstrated the greatest binding energy, signifying the most intense interaction between the Pt atom at the TW spot and the WTe_2_ layer. As a result, the Pt-WTe_2_ configuration in Figure 1b shows improved stability relative to those in Figure 1a,c, aligning with Xu’s findings [54]. It is established that the Pt-Te bond spans a length of 2.58 Å.

**Figure 3:**
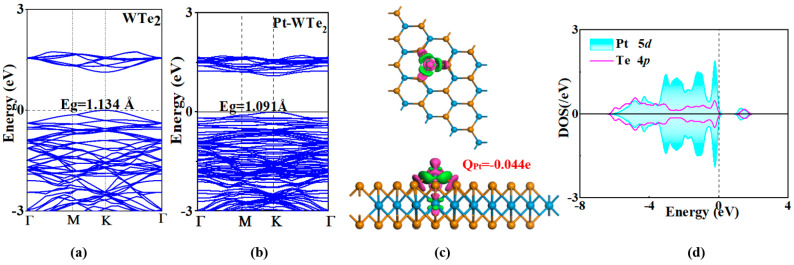
(**a**) Band structure of WTe_2_ monolayer; (**b**) band structure of Pt-WTe_2_; (**c**) 3D view of CDD of Pt atom-decorated WTe_2_; (**c**) density of states of the key atoms (Pt and Te atom).

Hirshfeld’s study revealed that the Pt adatom in the Pt-decoration procedure exhibited robust electron-accepting characteristics due to its negative charge of −0.044 e. Consequently, the Pt adatom led to a total loss of 0.044 e in the WTe_2_ monolayer, underscoring the advantageous electropositivity of the W and Te atoms in this setup [55]. Figure 3a illustrates the 3D depiction of Pt-WTe_2’_s CDD. The CDD analysis showed electron depletion encasing the Pt dopant and electron accumulation around the Pt-Te bonds, reinforcing the electron-attracting characteristic of the Pt adatom.

Figure 3b,c displays the BS for both the unaltered and Pt-adorned WTe_2_ monolayer, including the orbital DOS of crucial atoms, to facilitate comprehension of the electronic characteristics of the WTe_2_ monolayer with Pt decoration. An was of the BS in both the unaltered and Pt-WTe_2_ monolayer revealed that the K point is where both the highest valence band and the lowest conduction band were situated. The noted observation, coupled with a 1.127 eV band gap, indicates the inherent semiconductor properties of the unaltered WTe_2_ monolayer. Regarding the Pt-WTe_2_ monolayer, both the highest valence and lowest conduction bands were situated at the k point, suggesting that the Pt alteration barely affected the WTe_2_ monolayer’s direct semiconductor characteristics. Furthermore, the Pt-WTe_2_ monolayer exhibited a band gap of 1.091 eV, marginally less than the unaltered WTe_2_ monolayer. Additional DOS analysis uncovered a significant intersection between the Pt 5d and Te 4p orbital within the energy spectrum of −6~0 eV to 1.8 eV, suggesting a robust orbital interplay between the Pt and Te atom. This discovery validates the potent binding impact caused by the Pt alteration on WTe_2’_s surface.

### 3.2. Adsorption of CO and C_2_H_4_ on Pristine WTe_2_ and Pt-WTe_2_

**Figure 4:**
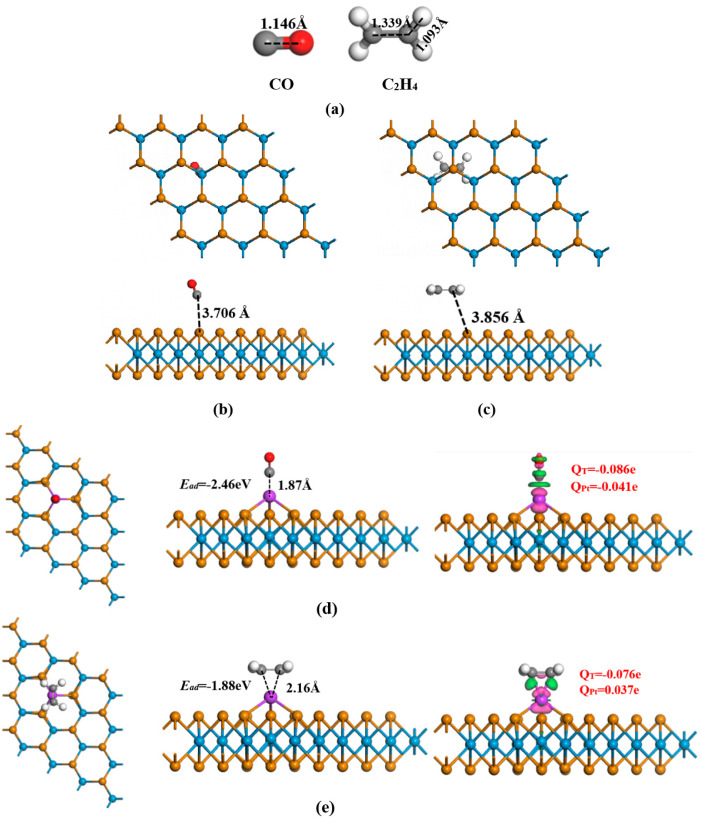
(**a**) Gases. Adsorption structures of CO and C_2_H_4_ on pristine WTe_2_: (**b**) CO system; (**c**) C_2_H_4_ system. Adsorption structures of CO and C_2_H_4_ on Pt-decorated WTe_2_: (**d**) CO system; (**e**) C_2_H_4_ system.

Following the identification of a single Pt atom’s doping site, our next step was to examine how CO and C_2_H_4_ adsorb on both unaltered WTe_2_ and Pt-WTe_2_. Figure 1c and Figure 4a display the atoms in question and the refined configurations of CO and C_2_H_4_, respectively. The bond lengths of C-O, C-H, and C=C have been determined to be 1.146 Å, 1.093 Å, and 1.339 Å, respectively. Post geometric optimization, the adsorption patterns of unaltered WTe_2_ on CO and C_2_H_4_ are depicted in Figure 4b,c. In the WTe_2_ monolayer, the gap between CO’s C atom and the adsorption site was 3.71 Å, whereas in C_2_H_4_, it was 3.86 Å from the WTe_2_ monolayer’s adsorption site. Similarly, the estimated adsorption energies for CO and C_2_H_4_ were −0.50 eV and −0.55 eV, in that order. Furthermore, the exchange of charge between CO and WTe_2_, and C_2_H_4_ and WTe_2_, was determined to be −0.017 e and −0.033 e, in that order. The data suggest a comparable binding affinity between WTe_2_ and C_2_H_4_, akin to that between WTe_2_ and CO.

The adsorption structures of Pt-WTe_2_ with CO and C_2_H_4_, following geometric optimization, are presented in Figure 4d,e. Table 1 displays the calculated adsorption energies of CO and C_2_H_4_, alongside the charge transfer between the gas molecules and Pt-WTe_2_. Notably, the adsorption energy of CO surpassed that of C_2_H_4_, and WTe_2_ had better adsorption performance compared with the adsorption characteristics of GeTe, MoTe_2_, and HfSe_2_ adsorbing CO and C_2_H_4_ respectively from the perspective of adsorption energy. Comparing these energies with those of pristine WTe_2_ monolayer adsorbing gas molecules revealed an enhancement in adsorption energies upon the doping of single Pt atom. This enhancement suggests that the presence of single Pt atom enhanced the capability of WTe_2_ to adsorb gas molecules, particularly showing a significant improvement in the adsorption capacity for CO.

**Figure 5:**
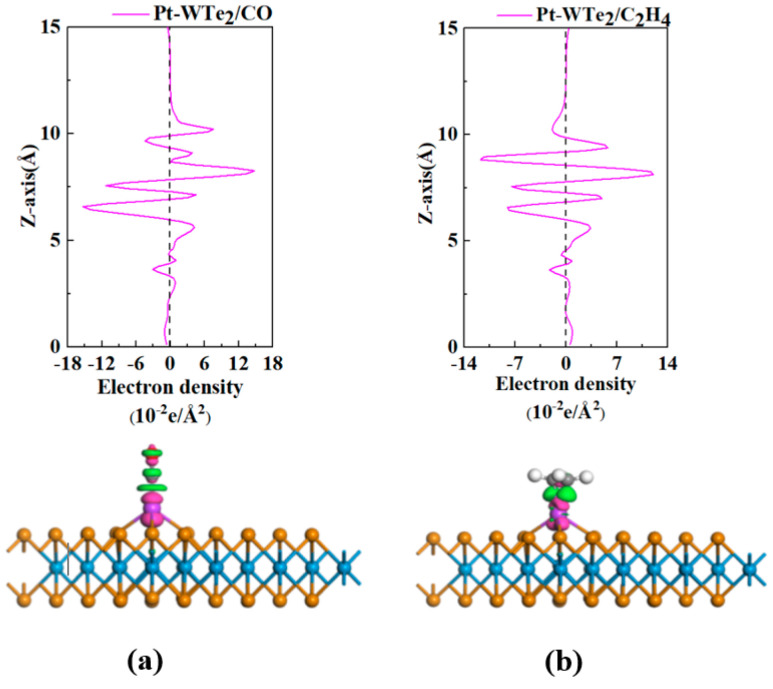
CDD of CO and C_2_H_4_ adsorbed on Pt atom-decorated WTe_2_ (the isosurface of 3D configurations is 0.01 e/Å^3^): (**a**) CO system; (**b**) C_2_H_4_ system.

For a deeper exploration of electron transfer, the arrangements of CDD were computed and are depicted in Figure 5. The depiction showed a three-dimensional representation of gas electron distribution absorbed by the Pt-WTe_2_ monolayer, with the isosurface value established at 0.01 e/Å^3^. Within the visual depiction, electron build-up is denoted by the pink regions, whereas the green zones represent electron reduction. Our findings emphasize that the pink areas mainly clustered near the Pt atom, whereas the green areas were concentrated around the gas molecule. The observed pattern indicates a significant transfer of charge between the gas and the Pt-WTe_2_ monolayer. A review of the z-axis projection in Figure 5 reveals that electron density readings exceeding 0 dominated when the gap between electrons and the Pt-WTe_2_ monolayer’s adsorption site surpassed 5 Å. For example, when Pt-WTe_2_ absorbs CO, the peak absolute electron density along the Z axis is around 15 e/Å^3^. Conversely, in the case of C_2_H_4_ adsorption, the measurement hovers near 11 e/Å^3^, suggesting a lesser degree of redistribution than CO adsorption on Pt-WTe_2_. Clearly, enhanced adsorption led to considerable redistribution of electrons.

### 3.3. Electronic Properties of Single Pt Atom-Doped WTe_2_ Before and After Adsorbing CO and C_2_H_4_

**Figure 6:**
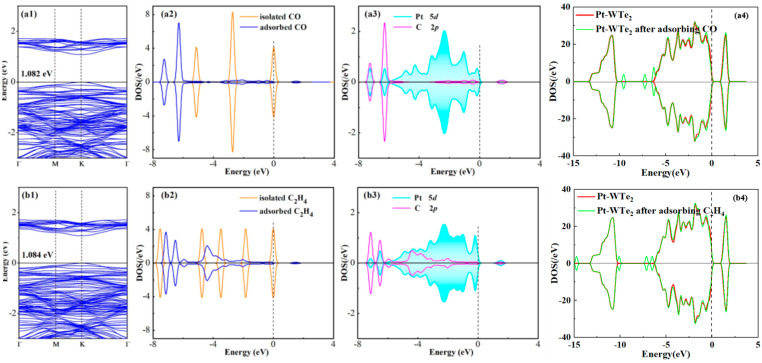
(**a1**) Band structure of Pt-WTe_2_ after adsorbing CO; (**b1**) band structure of Pt-WTe_2_ after adsorbing C_2_H_4_; (**a2**) molecular density of states of isolated CO and adsorbed CO; (**b2**) molecular density of states of isolated C_2_H_4_ and adsorbed C_2_H_4_; (**a3**) orbital density of states of atoms (Pt and C atom in the CO); (**b3**) orbital density of states of atoms (Pt and C atom in the C_2_H_4_); (**a4**) total density of states of Pt-WTe_2_ adsorbing CO (**b4**) total density of states of Pt-WTe_2_ adsorbing C_2_H_4_.

During the process of gas adsorption, charge transfer is crucial in altering the electronic characteristics of the Pt-WTe_2_ monolayer, underpinning its mechanism for detecting gas. Evaluating these detection traits involves a comparative study of Pt-WTe_2’_s electronic properties pre- and post-CO adsorption, with an emphasis on the BS and DOS. Figure 6(a1) and Figure 6(b1) illustrate the BS for the Pt-WTe_2_ monolayer following the adsorption of CO and C_2_H_4_, respectively. Analyzing the band structure distributions revealed that each gas system displayed the highest valence band and the lowest conduction band at the k point. This indicates that the Pt-WTe_2_ monolayer retained its direct semiconductor characteristics, even after gas adsorption, aligning with the Pt-WTe_2_ BS depicted in Figure 3b. Significant differences were noted in the bandgap of the Pt-WTe_2_ monolayer, showing a decrease ranging from 0.8% to 1.082 eV in the CO system and 0.6% to 1.084 eV in the C_2_H_4_ system. The changes observed are linked to the adsorption of gases, suggesting that the active electronic conditions of these gases greatly influence the system being adsorbed.

For an in-depth exploration of the chemical interplay between gases and the Pt-WTe_2_ monolayer, computational analyses were performed to ascertain the overall DOS (TDOS), the partial DOS (PDOS) of molecules absorbed, and the DOS of atomic orbitals. The outcomes achieved are illustrated in Figure 6(a2–a4,b2–b4). When examining the TDOS of Pt-WTe_2_ pre- and post-CO adsorption, distinct new peaks appeared near −9 eV and −7 eV. Additionally, a noticeable change in the DOS’s configuration is observed around −6 eV in the comparison between TDOS post-CO adsorption and pre-adsorption. In contrast, examining the TDOS pre- and post-C_2_H_4_ adsorption revealed minor peaks around −15 eV, −10 eV, −6 eV, and −7 eV. Generally, Pt-WTe_2’_s TDOS remains largely unchanged before and after gas adsorption. In the molecular DOS, the emergence of electronic states in gas species is clearly observable. In particular, the separated CO and C_2_H_4_ molecules demonstrated a division into smaller forms and underwent shifts to different extents. There was a notable intersection between the Pt 5d orbital and the CO molecule’s C 2p orbital at energy levels of −7.1 eV, −6.2 eV, −0.6 eV, and 1.8 eV. Likewise, there was a significant intersection with the C_2_H_4_ molecule’s C 2p orbital at energy levels of −7.2 eV, −6.5 eV, −5.8 eV, −4.0 eV, and 1.8 eV. The hybridization of these states underscores the intense interaction of atoms in orbit.

In summary, Pt-WTe_2_ exhibits distinct physical and chemical interactions with CO and C_2_H_4_. A comprehensive analysis of DOS indicated a more pronounced interaction between Pt-WTe_2_ and CO.

### 3.4. Sensing Properties of Pt-WTe_2_ to Detect CO and C_2_H_4_

Variations between two gas systems impact the Pt-WTe_2_ monolayer’s electronic bandgap. Therefore, it is plausible to expect changes in the electrical conductivity of the Pt-WTe_2_ monolayer, considering the mutual reliance of the materials’ electrical conductivity (*σ*) and bandgap (*Eg*). The electrical conductivity of Pt-WTe_2_ is mathematically represented as [58,59]:

(5) $$\sigma =e^{\left(-E_{g}/2KT\right)}$$

where Eg, K, and T symbolize the bandgap, Boltzmann’s constant (8.62 × 10^−5^ eV/K), and Kelvin temperature, in that order. Furthermore, the modified electrical conductivity might enable the Pt-WTe_2_ monolayer to act as a resistance-type gas detector, with the gas-sensing response (S) being computable as follows:

(6) $$S={(\sigma_{gas}}^{-1}-{\sigma_{pure}}^{-1})/{\sigma_{pure}}^{-1}\;$$

where $\sigma_{gas}$ and $\sigma_{pure}$ represent the electrical conductivity of the Pt-WTe_2_/gas system and the independent Pt-WTe_2_ monolayer, in that order.

Utilizing Equations (4) and (5) for calculations, the Pt-WTe_2_ monolayer exhibited a detection reaction of −0.16 and −0.13 for CO and C_2_H_4_, respectively, at ambient temperature. The findings indicate a notable detection reaction in both CO and C_2_H_4_ systems, signifying efficient detection abilities. Given this encouraging feature, we suggest that the Pt-WTe_2_ monolayer is highly promising as a resistance-type gas sensor in identifying CO and C_2_H_4_, exhibiting an advantageous sensitivity.

The WF method was utilized to determine the least amount of energy needed to emit electrons from the surface into the vacuum [60]. Assessing the variance in WF pre- and post-adsorption on the material’s surface allows for the extraction of relevant insights into the intricacies of electron emission from the material. The method to compute the WF is below:

(7) $$\Phi =E_{vacuum\;level}-E_{Fermi\;level}\;$$

**Figure 7:**
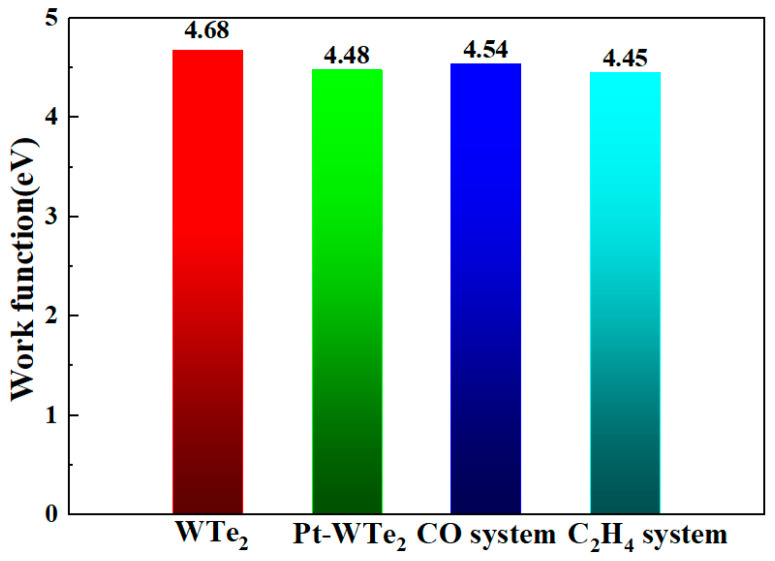
WF values of pristine WTe_2_, Pt-WTe_2_, Pt-WTe_2_ adsorbing CO, and Pt-WTe_2_ adsorbing C_2_H_4_.

An in-depth analysis of the WF values for both the unaltered and Pt-adorned WTe_2_ layers, along with the gas-absorbed systems, was conducted to thoroughly understand the response characteristic, with the WF values presented in Figure 7. Clearly, the Pt-WTe_2_ monolayer showed a reduced WF (4.48 eV) in contrast to the unaltered WTe_2_ monolayer (4.68 eV), suggesting that adorning with Pt may ease the process for the WTe_2_ monolayer to emit an electron in a vacuum. Additionally, the WF measurement for Pt-WTe_2_ absorbing CO rose to 4.54 eV, but fell to 4.45 eV when C_2_H_4_ was adsorbed. The notable alteration in the water flow (WF) of the Pt-WTe_2_ monolayer, marked by a 1.3% increase in CO adsorption and a 0.7% rise in C_2_H_4_ adsorption, underscored its robust reaction to CO. Put differently, the Pt−WTe_2_ single layer may act as the optimal gas detector for CO detection.

**Figure 8:**
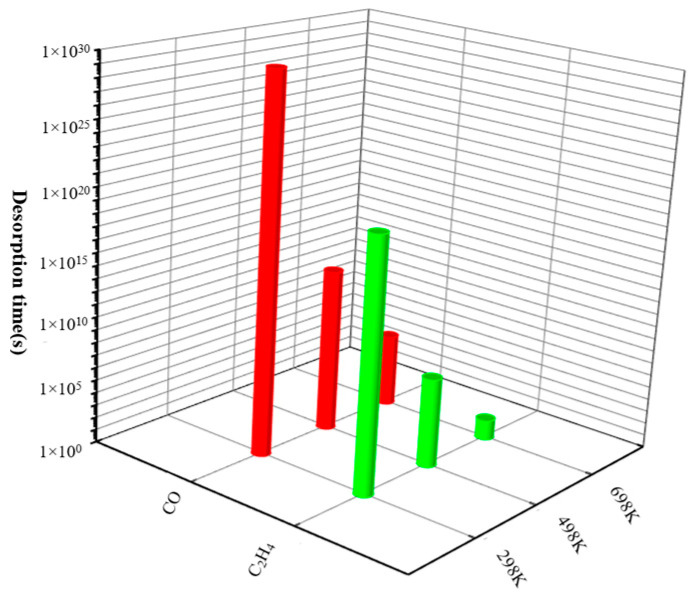
Desorption times of CO and C_2_H_4_ on Pt-WTe_2_ at different temperatures. (1 × 10^5^ denotes e^5^).

In gas sensing, the recovery property plays a critical role in the overall sensing performance. When the adsorption energy is too low, gas molecules struggle to interact effectively with the sensing material, resulting in a weak response. On the contrary, an excessively high adsorption energy can lead to the material being excessively bound to the gas molecules, hindering efficient detection and accurate recognition. Achieving satisfactory recovery properties involves finding a balance in both adsorption energy and desorption time. If the desorption time is excessively prolonged, it delays the sensing material’s ability to release gas molecules, impacting its responsiveness. Conversely, excessively rapid desorption may compromise the accuracy of detection. Therefore, it is crucial to maintain a reasonable range for both adsorption energy and desorption time to ensure efficient gas sensing. The Van’t-Hoff–Arrhenius formula, grounded in transition state theory, is utilized to ascertain the time of desorption, offering a crucial understanding of the gas molecules’ desorption kinetics from the detection material [61]:

(8) $$\tau =A^{-1}e^{(-E_{ads}/KT)}\;$$

where *A* and *E_ads_* are the apparent frequency and adsorption energy. The value of *A* is estimated to be 10^12^ *s*^−1^ [61,62]. The evaluation of desorption time for gases on Pt-WTe_2_ at various temperature are illustrated in Figure 8.

Our computations aim to ascertain the duration of desorption for gas molecules (CO, C_2_H_4_) on Pt-WTe_2_, spanning temperatures from 298 K to 498 K. Specifically, at 298 K, the desorption periods for CO and C_2_H_4_ on Pt-WTe_2_ were approximately 3.87 × 10^29^ s and 6.05 × 10^19^ s, respectively. Our illustration in Figure 8 reveals a notable decrease in gas molecule desorption times as the temperature increases. Notably, at the same temperature, the desorption time of CO was longer than that of C_2_H_4_. Extended desorption durations might indicate a risk of material overload, yet it is significant to note that using high-temperature desorption and surface cleansing can successfully reinstate the gas detection material’s initial efficiency for both CO and C_2_H_4_. These findings offer insights into strategies for maintaining and restoring the functionality of the gas-sensing material [63].

## 4. Conclusions

This research utilized a DFT-based approach to explore the adsorption patterns of CO and C_2_H_4_ on the Pt-WTe_2_ single layer. Investigating three doping locations revealed that the Pt atom favored adsorption over the W atom, possessing the highest binding energy of −3.12 eV. Owing to the adornment of Pt, there was a decrease in the bandgap of Pt-WTe_2_, leading to the appearance of multiple impurity states in the BS relative to the original WTe_2_ monolayer. Of the pair of gas molecules, CO exerted a significant impact on the adsorption of Pt-WTe_2_, exhibiting an adsorption energy of −2.46 eV. An examination of the DOS showed a significant intersection in the atomic orbitals of the Pt atom and the C atom in CO, as opposed to the Pt atom and C atom interaction in C_2_H_4_. This finding robustly suggests more intense interactions between Pt-WTe_2_ and CO compared with Pt-WTe_2_ and C_2_H_4_. Additionally, the alteration in the WF pre and post Pt-WTe_2_ adsorption of CO was more significant compared with the pre- and post-C_2_H_4_ adsorption on Pt-WTe_2_, indicating a more distinct reaction of Pt-WTe_2_ to CO. In light of these studies, Pt-WTe_2_ has surfaced as a viable material for sensing CO in dissolved gases found in transformer oil.

## Figures and Tables

**Table 1:** Adsorption energy, electron transfer, and adsorption distance of CO and C_2_H_4_ on pristine and Pt-decorated WTe_2_.

Adsorption Structure	*E_ads_* (eV)	Distance (Å)	Q_t_ (e)
MoTe_2_/CO [39]	−0.11	3.62	−0.002
MoTe_2_/C_2_H_4_	−0.16	5.67	0.001
GeTe/CO [56]	−0.27	2.99	0.011
GeTe/C_2_H_4_	−0.41	3.21	0.045
HfSe_2_/CO [57]	−0.16	4.06	0.004
HfSe_2_/C_2_H_4_	−0.34	3.39	0.028
WTe_2_/CO	−0.50	3.71	−0.017
WTe_2_/C_2_H_4_	−0.55	3.86	−0.033
Pt-WTe_2_/CO	−2.46	1.87	−0.086
Pt-WTe_2_/C_2_H_4_	−1.88	2.16	−0.076

## Data Availability

The data presented in this study are available in the article.
